# The complete mitochondrial genome of *Aporia crataegi* L. (Lepidoptera: Pieridae)

**DOI:** 10.1080/23802359.2019.1675550

**Published:** 2019-10-11

**Authors:** Yunchuan He, Xianmei Song, Xinpu Wang, Xin Gu

**Affiliations:** School of Agriculture, Ningxia University, Yinchuan, Ningxia, People’s Republic of China

**Keywords:** Completed mitochondrial genome, Lepidoptera, Pieridae, *Aporia crataegi* L

## Abstract

The complete mitochondrial genome of *Aporia crataegi* is 15,147 bp long, and consists of 13 protein-coding genes (PCGs), 2 ribosomal RNA genes, 22 transfer RNA (tRNA) genes, and a putative control region (GenBank accession No. MN371463). The nucleotide composition is significantly biased (A, G, C, and T is 39.66%, 7.30%, 11.41%, and 41.63%, respectively) with A + T contents of 81.29%. All PCGs are initiated by ATG, ATT, and ATC codons. Seven PCGs use a common stop codon of TAA, whereas the remaining six terminated with a single T. The phylogenetic relationships based on maximum-likelihood phylogenetic tree method showed that *A. crataegi* is closely related to *Aporia bieti*, *Mesapia peloria*, and *Aporia martineti*.

Black-veined white butterfly [*Aporia crataegi* (Linnaeus, 1758), Lepidoptera: Pieridae], is a well-known pest in orchards, as its larvae feed on different economically important plants (Hou and Bi 1986). The species’ distribution ranges from Europe to Eastern Asia (40–70°N), including Japan (Tolman and Lewington 2008). The species is one of the endangered species in South Korea, but it is abundantly found in circumferential countries, such as China, Japan, and Mongolia (Jeong et al. 2012). In the past, severe outbreaks of the species have been reported (Jure et al. 2017). The specimen of *A. crataegi* used in this study were collected from Yinchuan, Ningxia Province, China (38°29′59″N, 106°8′9″E), and now the specimens are stored in the Insect Herbarium, School of Agriculture, Ningxia University (SANXU, voucher number: SFD201905-01).

The complete sequence of *A. crataegi* was 15,147 bp in length with A + T content of 81.29% (GenBank accession No. MN371463), containing 13 protein coding genes (PCGs), 22 transfer RNAs (tRNA), 2 ribosomal RNAs (rRNA), and a putative control region (CR). The gene order and organization of *A. crataegi* are consistent with those of putative ancestor of insects (Boore 1999). The nucleotide composition of the mitogenome of *A. crataegi* is significantly biased (A,G,C and T is 39.66%, 7.30%,11.41%,and 41.63%, respectively) with A + T contents of 81.29%. Due to the structural role of the A + T-rich region, such as the initiation of replication in insect mitogenomes, several conserved sequences in the A + T-rich region have been proposed, particularly in Lepidoptera (Salvato et al. 2008). The AT-skew and GC-skew of this genome were −0.024 and −0.220, respectively. Twenty-three genes were oriented on the L-strand, whereas the others were transcribed on the H-strand.

The *A. crataegi* mitogenome harbours a 69 bp of repeated sequences in 8 regions. The longest overlap is 40 bp in length and located between *tRNA-Leu^(CUN)^* and *16S rRNA*. This mitogenome has a total of 95 bp intergenic spacer sequences, which is made up of 12 regions in the range from 1 to 49 bp. The largest intergenic spacer sequence of 49 bp is located between *tRNA-Gln* and *ND2*. The control region was located in CR genes with a length of 362 bp, and the A + T content was 81.02%. The length of these tRNAs ranged from 60 bp (tRNA-Met) to 71 bp (tRNA-Lys), A + T content ranged from 70.00% (*tRNA-Met*) to 92.54% (*tRNA-Glu*). Two rRNAs (*16S rRNA* and *12S rRNA*) are located between *tRNA-Leu^(CUN)^* and *tRNA-Val*, and between *tRNA-Val* and the control region, respectively. The *16S rRNA* was 1369 bp in length with A + T content of 84.81%, and the *12S rRNA* was 777 bp in length with A + T content of 85.71%.

The initial codons for 13 PCGs of *A. crataegi* were the canonical putative start codons ATN (ATG for *COI-III*, *ATP6*, *ND4*, *ND4L*, *CYTB*, and *ND1*; ATT for *ND2*, *ND3*, and *ND5*; ATC for *ATP8* and *ND6*). The typical termination codon (TAA) occurs in 7 PCGs, and the remaining PCGs including *ND2*, *COI*, *COII*, *ND3*, *ND4*, and *ND5* were terminated with a single T. Based on the concatenated amino acid sequences of 13 PCGs, the maximum-likelihood (ML) method was used to construct the phylogenetic relationship of *A. crataegi* with 19 Pieridae species. The result showed that *A. crataegi* is closely related to *Aporia bieti*, *Mesapia peloria*, and *Aporia martineti* (Figure 1).

**Figure 1.**
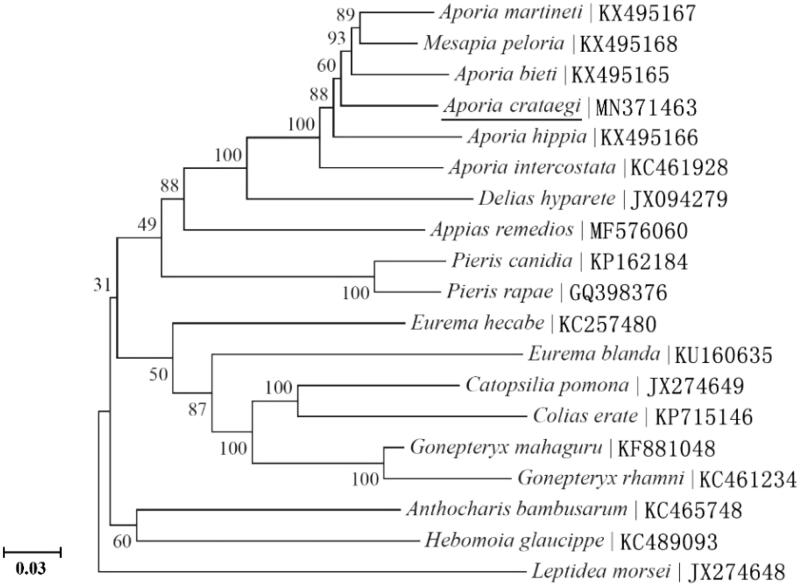
Phylogeny of 19 Pieridae species based on the maximum-likelihood (ML) analysis of 13 mitochondrial protein-coding genes. The best-fit nucleotide substitution model is ‘GTR + G + I’. The support values next to the nodes are based on 1000 bootstrap replicates. Gene Bank accession numbers of each species are listed in the tree.

## Nucleotide sequence accession number

GenBank accession number is MN371463.
